# Supplementary material for: Recovery of neuropsychological function following abstinence from alcohol in adults diagnosed with an alcohol use disorder: Systematic review of longitudinal studies
Source: PLoS One. 2024 Jan 2;19(1):e0296043. doi: 10.1371/journal.pone.0296043 (PMC10760842; doi:10.1371/journal.pone.0296043)
Supplement: S1 Table — (PDF) [file pone.0296043.s001.pdf]

PRISMA 2020 for Abstracts Checklist

Recovery of neuropsychological function following abstinence from alcohol in adults diagnosed with an Alcohol Use Disorder: Systematic review of longitudinal studies

Anna Powell, Harry Sumnall, Jessica Smith, Rebecca Kuiper, Catharine Montgomery

S1 Table. PRISMA 2020 abstracts checklist

Section and Topic	Item #	Checklist item	Reported (Yes/No)
**TITLE**			
Title	1	Identify the report as a systematic review.	Yes
**BACKGROUND**			
Objectives	2	Provide an explicit statement of the main objective(s) or question(s) the review addresses.	Yes
**METHODS**			
Eligibility criteria	3	Specify the inclusion and exclusion criteria for the review.	Yes
Information sources	4	Specify the information sources (e.g. databases, registers) used to identify studies and the date when each was last searched.	Yes
Risk of bias	5	Specify the methods used to assess risk of bias in the included studies.	Yes
Synthesis of results	6	Specify the methods used to present and synthesise results.	Yes
**RESULTS**			
Included studies	7	Give the total number of included studies and participants and summarise relevant characteristics of studies.	Yes
Synthesis of results	8	Present results for main outcomes, preferably indicating the number of included studies and participants for each. If meta-analysis was done, report the summary estimate and confidence/credible interval. If comparing groups, indicate the direction of the effect (i.e. which group is favoured).	Yes
**DISCUSSION**			
Limitations of evidence	9	Provide a brief summary of the limitations of the evidence included in the review (e.g. study risk of bias, inconsistency and imprecision).	Yes
Interpretation	10	Provide a general interpretation of the results and important implications.	Yes
**OTHER**			
Funding	11	Specify the primary source of funding for the review.	N/A
Registration	12	Provide the register name and registration number.	Yes
