# Supplementary material for: Recovery of neuropsychological function following abstinence from alcohol in adults diagnosed with an alcohol use disorder: Systematic review of longitudinal studies
Source: PLoS One. 2024 Jan 2;19(1):e0296043. doi: 10.1371/journal.pone.0296043 (PMC10760842; doi:10.1371/journal.pone.0296043)
Supplement: S3 Table — (PDF) [file pone.0296043.s003.pdf]

Recovery of neuropsychological function following abstinence from alcohol in adults diagnosed with an Alcohol Use Disorder: Systematic review of longitudinal studies

Anna Powell, Harry Sumnall, Jessica Smith, Rebecca Kuiper, Catharine Montgomery

S3 Table. Systematic search strategies for APA PsycInfo, EBSCO MEDLINE, CINAHL, and Web of Science

**Source(s)**	**Search Strategy**
**APA PsycInfo**, accessed via https://www.proquest.com/	i #1 ab(alcoholism OR alcoholic*)<br>ii #2 ab(alcohol OR drinking) AND ab(abus* OR addict* OR dependen* OR disorder* OR harmful)<br>iii #3 #1 OR #2<br>iv #4 ab(recover* OR abstinen* OR sober OR treatment)<br>v #5 #3 AND #4<br>vi #6 (cogniti* OR neuropsycholog*OR executive)<br>vii #7 ti(learning OR attention OR orientation OR switching OR shifting OR updating OR flexibility OR initiating OR motor OR planning OR “problem solving” OR “functional ability” OR “decision making” OR “time manag*” OR inhibit* OR monitor* OR “goal directed” OR “mental process*” OR memory OR dysexecutive OR intelligence OR IQ OR gait OR posture OR balance OR propriocept* OR “emotional function*” OR “emotion recognition” OR “emotional processing” OR language OR sensory OR perception OR vibrotactile OR visuospatial OR spatial OR "reaction time" OR "processing speed" OR "temporal order judgement" OR "amplitude discrimination" OR "duration discrimination" OR coordination)<br>viii #8 #6 OR #7<br>ix #9 #5 AND #8<br>x #10 ab(cohort OR prospective OR longitudinal OR “follow up*” OR retrospective OR “repeated measures” or timepoin* or “time poin*”))<br>xi #11 #9 AND #10<br>xii Filters – 1999-2022
**MEDLINE (EBSCO)** and **CINAHL**, accessed separately via https://web.a.ebscohost.com	i S1 (MH “Alcohol-Related Disorders+”)<br>ii S2 (MH “Alcoholism”)<br>iii S3 AB (alcohol OR drinking)<br>iv S4 AB (abus* OR addict* OR dependen* OR disorder* OR harmful)<br>v S5 S3 AND S4<br>vi S6 S1 OR S2 OR S5

	vii S7 AB recover* OR abstinen* OR sober OR treatment<br>viii S8 S6 AND S7<br>ix S9 (MH “Cognition Disorders+”)<br>x S10 (MH “Cognition+”)<br>xi S11 TX (cogniti* OR neuropsycholog*OR executive)<br>xii S12 TI learning OR attention OR orientation OR switching OR shifting OR updating OR flexibility OR initiating OR motor OR planning OR “problem solving” OR “functional ability” OR “decision making” OR “time manag*” OR inhibit* OR monitor* OR “goal directed” OR “mental process*” OR memory OR dysexecutive OR intelligence OR IQ OR gait OR posture OR balance OR propriocept* OR “emotional function*” OR “emotion recognition” OR “emotional processing” OR language OR sensory OR perception OR vibrotactile OR visuospatial OR spatial OR "reaction time" OR "processing speed" OR "temporal order judgement" OR "amplitude discrimination" OR "duration discrimination" OR coordination<br>xiii S13 (MH “Task Performance and Analysis+”)<br>xiv S14 (MH “Psychological Tests+”)<br>xv S15 S9 OR S10 OR S11 OR S12 OR S13 OR S14<br>xvi S16 S8 AND S15<br>xvii S17 AB cohort OR prospective OR longitudinal OR “follow up*” OR retrospective OR “repeated measures” OR 2timepoint* OR “time poin*”<br>xviii S18 S16 AND S17<br>xix Filters – 1999-2022, middle aged: 45-64 years, adult: 19-44 years, adult: 19+ years, adolescent: 13-18 years, young adult: 19-24 years
**Web of Science Core Collection**; accessed via https://www.webofscience.com/wos/	i #1 AB=(alcoholism OR alcoholic*)<br>ii #2 AB=(alcohol OR drinking)<br>iii #3 AB=(abus* OR addict* OR dependen* OR disorder* OR harmful)<br>iv #4 #2 AND #3<br>v #5 #1 OR #4<br>vi #6 AB=(recover* OR abstinen* OR sober OR treatment)<br>vii #7 #5 AND #6<br>viii #8 ALL=(cogniti* OR neuropsycholog*OR executive)<br>ix #9 TI=(learning OR attention OR orientation OR switching OR shifting OR updating OR flexibility OR initiating OR motor OR planning OR “problem solving” OR “functional ability” OR “decision making” OR “time manag*” OR inhibit* OR monitor* OR “goal directed” OR “mental process*” OR memory OR dysexecutive OR intelligence OR IQ OR gait OR posture OR balance OR propriocept* OR “emotional function*” OR “emotion

	recognition” OR “emotional processing” OR language OR sensory OR perception OR vibrotactile OR visuospatial OR spatial OR "reaction time" OR "processing speed" OR "temporal order judgement" OR "amplitude discrimination" OR "duration discrimination" OR coordination)<br>x #10 #8 OR #9<br>xi #11 #7 AND #10<br>xii #12 AB=(cohort OR prospective OR longitudinal OR “follow up*” OR retrospective OR “repeated measures” or timepoin* “time poin*”)<br>xiii #13 #10 AND #12<br>xiv Filters – 2022 OR 2021 OR 2020 OR 2019 OR 2018 OR 2017 OR 2016 OR 2015 OR 2014 OR 2012 OR 2011 OR 2009 OR 2010 OR 2008 OR 2007 OR 2006 OR 2005 OR 2004 OR 2003 OR 2002 OR 2001 OR 2000 OR 1999
