# Supplementary material for: Recovery of neuropsychological function following abstinence from alcohol in adults diagnosed with an alcohol use disorder: Systematic review of longitudinal studies
Source: PLoS One. 2024 Jan 2;19(1):e0296043. doi: 10.1371/journal.pone.0296043 (PMC10760842; doi:10.1371/journal.pone.0296043)
Supplement: S4 Table — (PDF) [file pone.0296043.s004.pdf]

Recovery of neuropsychological function following abstinence from alcohol in adults diagnosed with an Alcohol Use Disorder: Systematic review of longitudinal studies

Anna Powell, Harry Sumnall, Jessica Smith, Rebecca Kuiper, Catharine Montgomery

S4 Table. Papers excluded at the full-text screening stage, with reason for exclusion

Study	Reason for exclusion
	Initial database search
Witkiewitz et al. (2018)	Exposure (DSM-III-IV diagnosis)
Wilhelm et al. (2005)	Exposure (first follow-up earlier than 2 weeks of abstinence)
Mulhauser et al. (2018)	Population (alcohol not primary substance, comorbid SUDs)
Durazzo et al. (2007)	Exposure (baseline too late – after a month of abstinence)
Durazzo and Meyerhoff (2020)	Exposure (baseline too late – after a month of abstinence)
Rupp et al. (2021)	Exposure (baseline too late – after a month of abstinence)
Fein and Greenstein (2013)	Exposure (baseline too late – after a month of abstinence)
E. V. Sullivan et al. (2000)	Exposure (DSM-III-R diagnosis)
Sugarman et al. (2014)	Exposure (DSM-III-R diagnosis)
Witkiewitz et al. (2019)	Exposure (DSM-III-IV diagnosis)
Wilhelm et al. (2006)	Exposure (first follow-up earlier than 2 weeks of abstinence)
Bates et al. (2005)	Exposure (DSM-III-R diagnosis)
Dingwall et al. (2011)	Exposure (diagnosis unclear and no response from author)
Rourke and Grant (1999)	Exposure (DSM-III or DSM-III-R diagnosis)
Mann et al. (1999)	Exposure (DSM-III-R diagnosis)
Rosenbloom et al. (2007)	Exposure (baseline too late – after a month of abstinence)
Powell et al. (2021)	Exposure (first follow-up earlier than 2 weeks of abstinence)
Durazzo, Gazdzinski, et al. (2006)	Comparator (though abstinence duration was collected, was not statistically assessed as a comparator)
Mon et al. (2013)	Comparator (though abstinence duration was collected, was not statistically assessed as a comparator)
Gómez-Bujedo et al. (2022)	Comparator (though abstinence duration was collected, was not statistically assessed as a comparator)
Bruijnen et al. (2021)	Comparator (AUD vs ARBI)
Hoefer et al. (2014)	Comparator (despite including controls, these were not statistically compared to, and no regression analysis used to assess impact of abstinence duration)
Mon et al. (2009)	Comparator (despite including controls, these were not assessed regarding neuropsychological function, and no regression analysis used to assess impact of abstinence duration)
Bartsch et al. (2007)	Comparator (despite including controls, these were not statistically compared to, and no regression analysis used to assess impact of abstinence duration)
Maillard et al. (2020)	Comparator (not suitable)
Young et al. (2018)	Outcome (neuropsychological function not used in analyses as outcome)
Thorberg et al. (2016)	Outcome (not a neuropsychological function)
Zorlu et al. (2013)	Study design (cross-sectional)
Dawson and Grant (2000)	Study design (cross-sectional)

Horton and Roberts (2001)	Study design (cross-sectional)
Le Berre et al. (2016)	Study design (cross-sectional)
Ahluwalia et al. (2015)	Study design (neuropsychological function only assessed cross-sectionally)
Wilson et al. (2016)	Study design (analysis type)
Durazzo et al. (2013)	Study design (cross-sectional)
Meyerhoff and Durazzo (2020)	Study design (individuals who remained abstinent were not assessed longitudinally, only those who were non abstinent)
Handsearching round one	
Roseribloom et al. (2004)	Exposure (baseline too late – after a month of abstinence)
Bates et al. (2009)	Exposure (DSM-III-R diagnosis)
Moriyama et al. (2006)	Exposure (DSM-III-R diagnosis)
Ambekar and Goyal (2017)	Comparator (abstinence duration impact not assessed using regression analyses)
Demirakca et al. (2011)	Outcome (not neuropsychological function)
Gazdzinski et al. (2005)	Outcome (not neuropsychological function)
van Eijk et al. (2012)	Outcome (not neuropsychological function)
Gazdzinski et al. (2010)	Outcome (not neuropsychological function)
Davies et al. (2005)	Study design (cross-sectional)
Fein et al. (2006)	Study design (cross-sectional)
Naim-Feil et al. (2014)	Study design (cross-sectional)
Vollstädt-Klein et al. (2009)	Study design (cross-sectional)
Agartz et al. (2003)	Study design (neuropsychological function only assessed once)
Kornreich et al. (2001)	Study design (cross-sectional)
E. Sullivan et al. (2000)	Study design (cross-sectional)
Bjork et al. (2004)	Study design (cross-sectional)
Beatty et al. (2000)	Study design (cross-sectional)
Durazzo, Rothlind, et al. (2006)	Study design (cross-sectional)
Rosenbloom et al. (2005)	Study design (cross-sectional)
Noël et al. (2001)	Study design (cross-sectional)
Pitel et al. (2007)	Study design (cross-sectional)
Zinn et al. (2004)	Study design (cross-sectional)
Fama et al. (2006)	Study design (cross-sectional)
Handsearching round two	
Chanraud et al. (2007)	Study design (cross-sectional)
Duka et al. (2003)	Study design (cross-sectional)
Sullivan et al. (2002)	Study design (cross-sectional)
Tedstone and Coyle (2004)	Study design (cross-sectional)
Mlinarics et al. (2009)	Language (Hungarian)
Reviewer suggestions	
Luquiens et al. (2019)	Population (alcohol not primary substance, comorbid SUDs)

References

Agartz, I., Brag, S., Franck, J., Hammarberg, A., Okugawa, G., Svinhufvud, K., & Bergman, H. (2003). MR volumetry during acute alcohol withdrawal and abstinence: a descriptive study. *Alcohol and Alcoholism*, *38*(1), 71-78. https://doi.org/10.1093/alcalc/agg020

Ahluwalia, V., Wade, J. B., Moeller, F. G., White, M. B., Unser, A. B., Gavis, E. A., . . . Bajaj, J. S. (2015). The etiology of cirrhosis is a strong determinant of brain reserve: A multimodal magnetic resonance imaging study. *Liver Transplantation*, *21*(9), 1123-1132. https://doi.org/10.1002/lt.24163

Ambekar, P., & Goyal, S. (2017). Cognitive impairment and effects of abstinence on cognition in patients with alcohol dependence syndrome. *MedPulse – International Journal of Psychology*, *4*(1), 13-21.

Bartsch, A. J., Homola, G., Biller, A., Smith, S. M., Weijers, H.-G., Wiesbeck, G. A., . . . Bendszus, M. (2007). Manifestations of early brain recovery associated with abstinence from alcoholism. *Brain*, *130*(1), 36-47. https://doi.org/10.1093/brain/awl303

Bates, M. E., Barry, D., Labouvie, E. W., Fals-Stewart, W., Voelbel, G., & Buckman, J. F. (2009). Risk factors and neuropsychological recovery in clients with alcohol use disorders who were exposed to different treatments. Annual Scientific Meeting of the Research Society on Alcoholism, 24th, Jun, 2001, Montreal, PQ, Canada; Preliminary results of this study were presented at the aforementioned conference and at the 25th Annual Scientific Meeting of the Research Society on Alcoholism, San Francisco, Califomia, June 2002.,

Bates, M. E., Voelbel, G. T., Buckman, J. F., Labouvie, E. W., & Barry, D. (2005). Short-term neuropsychological recovery in clients with substance use disorders. *Alcoholism, Clinical and Experimental Research*, *29*(3), 367-377. https://doi.org/10.1097/01.alc.0000156131.88125.2a

Beatty, W. W., Tivis, R., Stott, H. D., Nixon, S. J., & Parsons, O. A. (2000). Neuropsychological deficits in sober alcoholics: influences of chronicity and recent alcohol consumption. *Alcoholism: Clinical and Experimental Research*, *24*(2), 149-154. https://doi.org/10.1111/j.1530-0277.2000.tb04584.x

Bjork, J. M., Hommer, D. W., Grant, S. J., & Danube, C. (2004). Impulsivity in abstinent alcohol-dependent patients: relation to control subjects and type 1–/type 2–like traits. *Alcohol*, *34*(2-3), 133-150. https://doi.org/10.1016/j.alcohol.2004.06.012

Bruijnen, C. J. W. H., Walvoort, S. J. W., Dijkstra, B. A. G., de Jong, C. A. J., & Kessels, R. P. C. (2021). The Course of Cognitive Performance during Inpatient Treatment in Patients with Alcohol Use Disorder with No, Mild or Major Neurocognitive Disorders. *Alcohol and Alcoholism*, *56*(1), 89-100. https://doi.org/10.1093/alcalc/agaa100

Chanraud, S., Martelli, C., Delain, F., Kostogianni, N., Douaud, G., Aubin, H.-J., . . . Martinot, J.-L. (2007). Brain morphometry and cognitive performance in detoxified alcohol-dependents with preserved psychosocial functioning. *Neuropsychopharmacology*, *32*(2), 429-438.

Davies, S. J., Pandit, S. A., Feeney, A., Stevenson, B. J., Kerwin, R. W., Nutt, D. J., . . . Lingford-Hughes, A. (2005). Is there cognitive impairment in clinically 'healthy'abstinent alcohol dependence? *Alcohol and Alcoholism*, *40*(6), 498-503. https://doi.org/10.1093/alcalc/agh203

Dawson, L. K., & Grant, I. (2000). Alcoholics' initial organizational and problem-solving skills predict learning and memory performance on the Rey–Osterrieth Complex Figure. *Journal of the International Neuropsychological Society*, *6*(1), 12-19. https://doi.org/10.1017/s1355617700611025

Demirakca, T., Ende, G., Kämmerer, N., Welzel-Marquez, H., Hermann, D., Heinz, A., & Mann, K. (2011). Effects of alcoholism and continued abstinence on brain volumes in both genders.

Alcoholism: Clinical and Experimental Research, *35*(9), 1678-1685. https://doi.org/10.1111/j.1530-0277.2011.01514.x
Dingwall, K. M., Maruff, P., & Cairney, S. (2011). Similar profile of cognitive impairment and recovery for Aboriginal Australians in treatment for episodic or chronic alcohol use. *Addiction*, *106*(8), 1419-1426. https://doi.org/10.1111/j.1360-0443.2011.03434.x
Duka, T., Townshend, J. M., Collier, K., & Stephens, D. N. (2003). Impairment in cognitive functions after multiple detoxifications in alcoholic inpatients. *Alcoholism: Clinical and Experimental Research*, *27*(10), 1563-1572. https://doi.org/10.1097/01.ALC.0000090142.11260.D7
Durazzo, T. C., Gazdzinski, S., Rothlind, J. C., Banys, P., & Meyerhoff, D. J. (2006). Brain metabolite concentrations and neurocognition during short-term recovery from alcohol dependence: preliminary evidence of the effects of concurrent chronic cigarette smoking. *Alcoholism: Clinical and Experimental Research*, *30*(3), 539-551. https://doi.org/10.1111/j.1530-0277.2006.00060.x
Durazzo, T. C., & Meyerhoff, D. J. (2020). Cigarette smoking history is associated with poorer recovery in multiple neurocognitive domains following treatment for an alcohol use disorder. *Alcohol*, *85*, 135-143. https://doi.org/10.1016/j.alcohol.2019.12.003
Durazzo, T. C., Pennington, D. L., Schmidt, T. P., Mon, A., Abé, C., & Meyerhoff, D. J. (2013). Neurocognition in 1-month-abstinent treatment-seeking alcohol-dependent individuals: interactive effects of age and chronic cigarette smoking. *Alcoholism: Clinical and Experimental Research*, *37*(10), 1794-1803. https://doi.org/10.1111/acer.12140
Durazzo, T. C., Rothlind, J. C., Gazdzinski, S., Banys, P., & Meyerhoff, D. J. (2006). A comparison of neurocognitive function in nonsmoking and chronically smoking short-term abstinent alcoholics. *Alcohol*, *39*(1), 1-11. https://doi.org/10.1016/j.alcohol.2006.06.006
Durazzo, T. C., Rothlind, J. C., Gazdzinski, S., Banys, P., & Meyerhoff, D. J. (2007). Chronic smoking is associated with differential neurocognitive recovery in abstinent alcoholic patients: a preliminary investigation. *Alcoholism: Clinical and Experimental Research*, *31*(7), 1114-1127. https://doi.org/10.1111/j.1530-0277.2007.00398.x
Fama, R., Pfefferbaum, A., & Sullivan, E. V. (2006). Perceptual learning in detoxified alcoholic men: contributions from explicit memory, executive function, and age. *Alcoholism: Clinical and Experimental Research*, *28*(11), 1657-1665. https://doi.org/10.1097/01.ALC.0000145690.48510.DA
Fein, G., & Greenstein, D. (2013). Gait and balance deficits in chronic alcoholics: no improvement from 10 weeks through 1 year abstinence. *Alcoholism, Clinical and Experimental Research*, *37*(1), 86-95. https://doi.org/10.1111/j.1530-0277.2012.01851.x
Fein, G., Torres, J., Price, L. J., & Di Sclafani, V. (2006). Cognitive performance in long-term abstinent alcoholic individuals. *Alcoholism: Clinical and Experimental Research*, *30*(9), 1538-1544. https://doi.org/10.1111/j.1530-0277.2006.00185.x
Gazdzinski, S., Durazzo, T. C., & Meyerhoff, D. J. (2005). Temporal dynamics and determinants of whole brain tissue volume changes during recovery from alcohol dependence. *Drug and Alcohol Dependence*, *78*(3), 263-273. https://doi.org/10.1016/j.drugalcdep.2004.11.004
Gazdzinski, S., Durazzo, T. C., Mon, A., Yeh, P.-H., & Meyerhoff, D. J. (2010). Cerebral white matter recovery in abstinent alcoholics—a multimodality magnetic resonance study. *Brain*, *133*(4), 1043-1053. https://doi.org/10.1093/brain/awp343
Gómez-Bujedo, J., Lorca-Marín, J. A., Pérez-Moreno, P. J., Diaz Batanero, C., Fernández-Calderón, F., & Moraleda-Barreno, E. (2022). Changes in Drug-Related Implicit Associations during Substance Use Disorder Treatment: The Role of the Therapeutic Context. *Substance Use & Misuse*, *57*(2), 185-192. https://doi.org/10.1080/10826084.2021.1995755
Hoefer, M. E., Pennington, D. L., Durazzo, T. C., Mon, A., Abé, C., Truran, D., . . . Meyerhoff, D. J. (2014). Genetic and behavioral determinants of hippocampal volume recovery during abstinence from alcohol. *Alcohol*, *48*(7), 631-638. https://doi.org/10.1016/j.alcohol.2014.08.007

Horton, A. M., & Roberts, C. (2001). Demographic effects on the Trail Making test in alcohol abusers. *International Journal of Neuroscience*, *109*(3-4), 281-287. https://doi.org/10.3109/00207450108986539

Kornreich, C., Blairy, S., Philippot, P., Hess, U., Noël, X., Streel, E., . . . Verbanck, P. (2001). Deficits in recognition of emotional facial expression are still present in alcoholics after mid-to long-term abstinence. *Journal of studies on alcohol*, *62*(4), 533-542. https://doi.org/10.15288/jsa.2001.62.533

Le Berre, A. P., Müller-Oehring, E. M., Kwon, D., Serventi, M. R., Pfefferbaum, A., & Sullivan, E. V. (2016). Differential compromise of prospective and retrospective metamemory monitoring and their dissociable structural brain correlates. *Cortex*, *81*, 192-202. https://doi.org/10.1016/j.cortex.2016.05.002

Luquiens, A., Rolland, B., Pelletier, S., Alarcon, R., Donnadieu-Rigole, H., Benyamina, A., . . . Perney, P. (2019). Role of patient sex in early recovery from alcohol-related cognitive impairment: women penalized. *Journal of Clinical Medicine*, *8*(6), 790. https://doi.org/10.3390/jcm8060790

Maillard, A., Poussier, H., Boudehent, C., Lannuzel, C., Vicente, A., Vabret, F., . . . Pitel, A.-L. (2020). Short-term neuropsychological recovery in alcohol use disorder: A retrospective clinical study. *Addictive Behaviors*, *105*, 106350. https://doi.org/10.1016/j.addbeh.2020.106350

Mann, K., Günther, A., Stetter, F., & Ackermann, K. (1999). Rapid recovery from cognitive deficits in abstinent alcoholics: A controlled test-retest study. *Alcohol and Alcoholism*, *34*(4), 567-574. https://doi.org/10.1093/alcalc/34.4.567

Meyerhoff, D. J., & Durazzo, T. C. (2020). Not all is lost for relapsers: relapsers with low WHO risk drinking levels and complete abstainers have comparable regional gray matter volumes. *Alcoholism: Clinical and Experimental Research*, *44*(7), 1479-1487. https://doi.org/10.1111/acer.14377

Mlinarics, R., Kelemen, O., Sefcsik, T., & Németh, D. (2009). Cognitive impairment in patients with alcoholism after long-term abstinence. *Neuropsychopharmacologia Hungarica: a Magyar Pszichofarmakologiai Egyesulet Lapja= Official Journal of the Hungarian Association of Psychopharmacology*, *11*(3), 135-139.

Mon, A., Durazzo, T. C., Gazdzinski, S., Hutchison, K. E., Pennington, D., & Meyerhoff, D. J. (2013). Brain-derived neurotrophic factor genotype is associated with brain gray and white matter tissue volumes recovery in abstinent alcohol-dependent individuals. *Genes, Brain, and Behavior*, *12*(1), 98-107. https://doi.org/10.1111/j.1601-183X.2012.00854.x

Mon, A., Durazzo, T. C., Gazdzinski, S., & Meyerhoff, D. J. (2009). The impact of chronic cigarette smoking on recovery from cortical gray matter perfusion deficits in alcohol dependence: longitudinal arterial spin labeling MRI. *Alcoholism, Clinical and Experimental Research*, *33*(8), 1314-1321. https://doi.org/10.1111/j.1530-0277.2009.00960.x

Moriyama, Y., Muramatsu, T., Kato, M., Mimura, M., & Kashima, H. (2006). Family history of alcoholism and cognitive recovery in subacute withdrawal. *Psychiatry and Clinical Neurosciences*, *60*(1), 85-89. https://doi.org/10.1111/j.1440-1819.2006.01464.x

Mulhauser, K., Weinstock, J., Ruppert, P., & Benware, J. (2018). Changes in neuropsychological status during the initial phase of abstinence in alcohol use disorder: neurocognitive impairment and implications for clinical care. *Substance Use & Misuse*, *53*(6), 881-890. https://doi.org/10.1080/10826084.2017.1408328

Naim-Feil, J., Fitzgerald, P. B., Bradshaw, J. L., Lubman, D. I., & Sheppard, D. (2014). Neurocognitive deficits, craving, and abstinence among alcohol-dependent individuals following detoxification. *Archives of Clinical Neuropsychology*, *29*(1), 26-37. https://doi.org/10.1093/arclin/act090

Noël, X., Paternot, J., Van der Linden, M., Sferrazza, R., Verhas, M., Hanak, C., . . . Pelc, I. (2001). Correlation between inhibition, working memory and delimited frontal area blood flow

measured by 99MTC–bicisate spect in alcohol–dependent patients. *Alcohol and Alcoholism*, *36*(6), 556-563. https://doi.org/10.1093/alcalc/36.6.556

Pitel, A. L., Beaunieux, H., Witkowski, T., Vabret, F., Guillery-Girard, B., Quinette, P., . . . Eustache, F. (2007). Genuine episodic memory deficits and executive dysfunctions in alcoholic subjects early in abstinence. *Alcoholism: Clinical and Experimental Research*, *31*(7), 1169-1178. https://doi.org/10.1111/j.1530-0277.2007.00418.x

Powell, A., Tommerdahl, M., Abbasi, Y., Sumnall, H., & Montgomery, C. (2021). A pilot study assessing the brain gauge as an indicator of cognitive recovery in alcohol dependence. *Human Psychopharmacology: Clinical and Experimental*, *36*(4), e2782. https://doi.org/10.1002/hup.2782

Rosenbloom, M. J., O'Reilly, A., Sassoon, S. A., Sullivan, E. V., & Pfefferbaum, A. (2005). Persistent cognitive deficits in community-treated alcoholic men and women volunteering for research: limited contribution from psychiatric comorbidity. *Journal of studies on alcohol*, *66*(2), 254-265. https://doi.org/10.15288/jsa.2005.66.254

Rosenbloom, M. J., Rohlfing, T., O'Reilly, A. W., Sassoon, S. A., Pfefferbaum, A., & Sullivan, E. V. (2007). Improvement in memory and static balance with abstinence in alcoholic men and women: Selective relations with change in brain structure. *Psychiatry Research: Neuroimaging*, *155*(2), 91-102. https://doi.org/10.1016/j.pscychresns.2006.12.019

Roseribloom, M. J., Pfefferbaum, A., & Sullivan, E. V. (2004). Recovery of short-term memory and psychomotor speed but not postural stability with long-term sobriety in alcoholic women. *Neuropsychology*, *18*(3), 589. https://doi.org/10.1037/0894-4105.18.3.589

Rourke, S. B., & Grant, I. (1999). The interactive effects of age and length of abstinence on the recovery of neuropsychological functioning in chronic male alcoholics: A 2-year follow-up study. *Journal of the International Neuropsychological Society*, *5*(3), 234-246. https://doi.org/10.1017/s1355617799533067

Rupp, C. I., Junker, D., Kemmler, G., Mangweth-Matzek, B., & Derntl, B. (2021). Do Social Cognition Deficits Recover with Abstinence in Alcohol-Dependent Patients? *Alcoholism, Clinical and Experimental Research*, *45*(2), 470-479. https://doi.org/10.1111/acer.14537

Sugarman, D. E., Kaufman, J. S., Trucco, E. M., Brown, J. C., & Greenfield, S. F. (2014). Predictors of drinking and functional outcomes for men and women following inpatient alcohol treatment. *American Journal on Addictions*, *23*(3), 226-233. https://doi.org/10.1111/j.1521-0391.2014.12098.x

Sullivan, E., Rosenbloom, M. J., & Pfefferbaum, A. (2000). Pattern of motor and cognitive deficits in detoxified alcoholic men. *Alcoholism: Clinical and Experimental Research*, *24*(5), 611-621. https://doi.org/10.1111/j.1530-0277.2000.tb02032.x

Sullivan, E. V., Fama, R., Rosenbloom, M. J., & Pfefferbaum, A. (2002). A profile of neuropsychological deficits in alcoholic women. *Neuropsychology*, *16*(1), 74. https://doi.org/10.1037/0894-4105.16.1.74

Sullivan, E. V., Rosenbloom, M. J., Lim, K. O., & Pfefferbaum, A. (2000). Longitudinal changes in cognition, gait, and balance in abstinent and relapsed alcoholic men: relationships to changes in brain structure. *Neuropsychology*, *14*(2), 178. https://doi.org/10.1037/0894-4105.14.2.178

Tedstone, D., & Coyle, K. (2004). Cognitive impairments in sober alcoholics: performance on selective and divided attention tasks. *Drug and Alcohol Dependence*, *75*(3), 277-286. https://doi.org/10.1016/j.drugalcdep.2004.03.005

Thorberg, F. A., Young, R. M., Sullivan, K. A., Lyvers, M., Hurst, C. P., Connor, J. P., . . . Feeney, G. F. (2016). A longitudinal mediational study on the stability of alexithymia among alcohol-dependent outpatients in cognitive–behavioral therapy. *Psychology of Addictive Behaviors*, *30*(1), 64. https://doi.org/10.1037/adb0000135

van Eijk, J., Demirakca, T., Frischknecht, U., Hermann, D., Mann, K., & Ende, G. (2012). Rapid Partial Regeneration of Brain Volume During the First 14 Days of Abstinence from Alcohol.

Alcoholism: Clinical and Experimental Research, *37*(1), 67-74. https://doi.org/10.1111/j.1530-0277.2012.01853.x
Vollstädt-Klein, S., Loeber, S., Von der Goltz, C., Mann, K., & Kiefer, F. (2009). Avoidance of alcohol-related stimuli increases during the early stage of abstinence in alcohol-dependent patients. *Alcohol & Alcoholism*, *44*(5), 458-463. https://doi.org/10.1093/alcalc/agp056
Wilhelm, J., Bayerlein, K., Hillemacher, T., Reulbach, U., Frieling, H., Kromolan, B., . . . Bleich, S. (2006). Short-term cognition deficits during early alcohol withdrawal are associated with elevated plasma homocysteine levels in patients with alcoholism. *Journal of Neural Transmission*, *113*, 357-363. https://doi.org/10.1007/s00702-005-0333-1
Wilhelm, J., von Ahsen, N., Frieling, H., Hillemacher, T., Bayerlein, K., Bönsch, D., . . . Bleich, S. (2005). Apolipoprotein E4 genotype is not associated with short-term cognition deficits during alcohol withdrawal. *Alcohol*, *37*(3), 151-156. https://doi.org/10.1016/j.alcohol.2006.01.002
Wilson, A. D., Bravo, A. J., Pearson, M. R., & Witkiewitz, K. (2016). Finding success in failure: using latent profile analysis to examine heterogeneity in psychosocial functioning among heavy drinkers following treatment. *Addiction*, *111*(12), 2145-2154. https://doi.org/10.1111/add.13518
Witkiewitz, K., Kirouac, M., Roos, C. R., Wilson, A. D., Hallgren, K. A., Bravo, A. J., . . . Maisto, S. A. (2018). Abstinence and low risk drinking during treatment: Association with psychosocial functioning, alcohol use, and alcohol problems 3 years following treatment. *Psychology of addictive behaviors : journal of the Society of Psychologists in Addictive Behaviors*, *32*(6), 639-646. https://doi.org/10.1037/adb0000381
Witkiewitz, K., Wilson, A. D., Pearson, M. R., Montes, K. S., Kirouac, M., Roos, C. R., . . . Maisto, S. A. (2019). Profiles of recovery from alcohol use disorder at three years following treatment: can the definition of recovery be extended to include high functioning heavy drinkers? *Addiction*, *114*(1), 69-80. https://doi.org/10.1111/add.14403
Young, S. Y., Kidd, M., van Hoof, J. J. M., & Seedat, S. (2018). Prognostic Value of Motor Timing in Treatment Outcome in Patients With Alcohol- and/or Cocaine Use Disorder in a Rehabilitation Program. *Frontiers in Psychology*, *9*, 1945. https://doi.org/10.3389/fpsyg.2018.01945
Zinn, S., Stein, R., & Swartzwelder, H. S. (2004). Executive functioning early in abstinence from alcohol. *Alcoholism: Clinical and Experimental Research*, *28*(9), 1338-1346.
Zorlu, N., Gelal, F., Kuserli, A., Cenik, E., Durmaz, E., Saricicek, A., & Gulseren, S. (2013). Abnormal white matter integrity and decision-making deficits in alcohol dependence. *Psychiatry Research: Neuroimaging*, *214*(3), 382-388. https://doi.org/10.1016/j.pscychresns.2013.06.014
